# Genomic Instability and Cytotoxicity Evaluation of Two Communities Exposed to Pesticides in the Mexicali Valley by the L-CBMN Assay

**DOI:** 10.3390/toxics11100807

**Published:** 2023-09-25

**Authors:** Balam Ruiz-Ruiz, Olivia Torres-Bugarin, Erika Zúñiga-Violante, Francisco Casillas-Figueroa, Roberto Luna-Vázquez-Gómez, Verónica Campos Gallegos, Ana Erika Ruiz-Arellano, María Evarista Arellano-García

**Affiliations:** 1 Laboratorio de Genotoxicología Ambiental, Facultad de Ciencias, Universidad Autónoma de Baja California, Ensenada 22860, Baja California, Mexico; bruiz@uabc.edu.mx (B.R.-R.); erikazunigav@gmail.com (E.Z.-V.); casillas.francisco@uabc.edu.mx (F.C.-F.); rluna@uabc.edu.mx (R.L.-V.-G.); campgveronica@gmail.com (V.C.G.); 2 Laboratorio de Evaluación de Genotóxicos, Medicina Interna II, Facultad de Medicina, Decanato de Ciencias de la Salud, Universidad Autónoma de Guadalajara, Zapopan 45129, Jalisco, Mexico; 3 Facultad de Ingeniería, Arquitectura y Diseño, Universidad Autónoma de Baja California, Ensenada 22860, Baja California, Mexico; erika.ruiz@uabc.edu.mx

**Keywords:** lacto-ovo vegetarian diet, genotoxicity, micronuclei, pesticides, genomic instability

## Abstract

The continuous biomonitoring of a population directly or indirectly exposed to pesticides could be an additional tool for decision makers to improve their health conditions. In this work, we performed biomonitoring on two groups of people from the Mexicali Valley who were continuously exposed to pesticides using the cytokinesis-block micronucleus cytome assay (L-CBMN) to evaluate cytotoxic and genotoxic damage in human peripheral blood lymphocytes. The study groups comprised 14 indigenous Cucapah with non-vegetarian habits (NV group) from Ejido el Mayor (32.12594°, −115.27265°) and 21 lacto-ovo vegetarian (LOV) persons from the Seventh-day Adventist Church of Ejido Vicente Guerrero (32.3961°, −115.14023°). The L-CBMN assay determines the nuclear division index (NDI), apoptosis, necrosis, micronuclei (MNs), nuclear buds (NBUDs), and nucleoplasmic bridges (NPBs). Our results show that, regardless of diet or daily habits, both the studied groups presented with cytogenotoxic damage compared with non-exposed pesticide individuals, without modifications to the nuclear division index. In the rest of the evaluated biomarkers, the NV group exhibited greater cytotoxic and genotoxic damage than the LOV group. Nevertheless, individuals practicing a lacto-ovo vegetarian diet (LOV) showed lower damage than those with non-vegetarian habits (NV), suggesting a better antioxidant response that helps decrease the genotoxic damage due to the enhanced intake of folates and antioxidants from a plant-based diet.

## 1. Introduction

Our daily activities expose us to countless substances that can harm our health. If this exposure occurs constantly, it can represent a significant risk for the population and future generations. In people whose main activity is agriculture, occupational exposure to different pesticides is widespread, and it may be related to a significant increase in chronic degenerative diseases such as cancer. The most affected populations are those of developing countries, with 40% of the total population working in the agricultural sector compared with 3% in industrialized countries [1,2]. However, the inhabitants of agricultural regions are also susceptible to health issues by non-occupational pesticide exposure due to soil and water pollution [3,4]. This phenomenon occurs worldwide and increases continuously due to the great demand for food.

In Mexico, particularly in the Mexicali Valley, agricultural activity is associated with high pesticide exposure due to increased human and cattle feed crop production [5,6]. Pesticides are a primary source of water and soil pollution, which impacts the health of living organisms, including humans. After exposure, pesticides can enter the body through oral, respiratory, and dermal routes [7]. Most pesticides can cause alterations in the genetic material and the possible development of some types of tumors. The inhabitants of this region include groups that could be particularly susceptible to genotoxicity by pesticide exposure due to their economic conditions or daily habits [8].

The Mexicali Valley is home to the Cucapah, an Indigenous group that has preserved its social, economic, political, and cultural structures [9]. Unfortunately, keeping their customs has left them impoverished and subjected to significant social marginalization, with little access to education, health, and essential services. The increase in temperature, decrease in rainfall, increasing use of water for crop production, and contamination of the water after its use limit the main Cucapah livelihood of fishing [10]. On the other hand, this area is also home to a large community of the Seventh-day Adventist Church, which advocates a vegetarian diet to its members and discourages consuming alcoholic beverages, tobacco, and illegal drugs [11]. Some studies suggest that the lifestyle of this community, particularly the dietary habits, could contribute to low DNA damage, lowering the risk of cancer, obesity, type 2 diabetes mellitus, asthma, and cardiovascular and neurodegenerative diseases [12,13,14,15,16].

According to the degree of animal products avoided, a person following a vegetarian diet could be sub-classified as a fruitarian, vegan, ovo-vegetarian, lacto-vegetarian, or lacto-ovo vegetarian (LOV) [17]. These diets are rich in folates, vitamins A, C, and E, and different carotenoids, all well-known antioxidant agents [17,18]. However, vegetarians could present with deficiencies in critical micronutrients such as high-quality proteins, iron, zinc, copper, calcium, and vitamins D and B_12_ and could result in an insufficient intake of sulfur-containing amino acids, affecting, in turn, the glutathione (GSH) production. A deficiency of these micronutrients, typically provided by animal sources, lowers an individual’s antioxidant defense, contributing to increased redox diseases [19,20,21].

The issue of controversial results about genetic damage found in vegetarians (Vs) and non-vegetarians (NVs) is a current topic [17,22,23,24]. Some results using human peripheral blood lymphocytes show no difference in the micronucleus frequency associated with genomic instability, and it is even noteworthy that some studies mention that the number of micronucleate lymphocytes is higher in Vs than in NVs [17,25,26]. However, other results show that vegetarians, compared to NVs, have a lower frequency of micronucleate reticulocytes [17,27].

Due to the controversial results, we must have the tools to carry out constant and low-cost biomonitoring of a population as large as possible in order to provide reliable and reproducible data. Nuclear damage to peripheral blood lymphocytes is a recognized warning signal for adverse health effects. The cytokinesis-block micronucleus cytome assay (L-CBMN) identifies the frequency of micronuclei (MNs), nuclear buds (NBUDs), and nucleoplasmic bridges (NPBs) on human lymphocytes obtained from peripheral blood samples (HPBLs) to assess the genotoxic risk and evaluates the nuclear division index (NDI), necrosis, and apoptosis as cytotoxic biomarkers [28,29]. The L-CBMN allows for the reasonable identification of genomic instability in vulnerable individuals using a relatively low-cost and easy-to-implement technique. Therefore, this study aims to identify the health risks and the possible contribution of lifestyle habits for two population groups with continuous direct or indirect exposure to significant amounts of pesticides in the Mexicali Valley by evaluating cytotoxic and genotoxic biomarkers.

## 2. Materials and Methods

### 2.1. Ethical Considerations

The study followed the Mexican Research Regulations and the Declaration of Helsinki. The Research and Bioethics Committee of the Autonomous University of Baja California approved the study protocol under registration number 5-031-074-07-001. Each participant was informed about the scope of the study, the handling of the confidentiality of the data provided, and gave their informed consent before sampling.

### 2.2. Study Area

**Figure 1:**
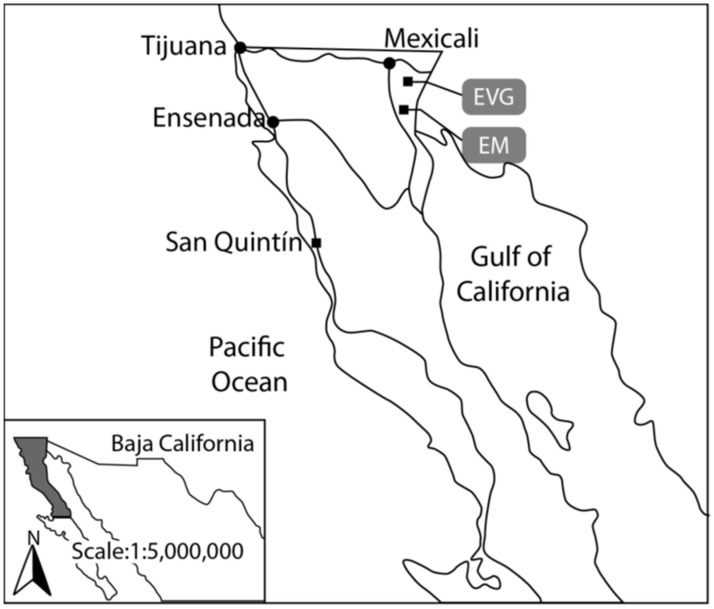
The Mexicali Valley in Baja California, Mexico. Located in northern Mexico, Ejido Vicente Guerrero (EVG) and Ejido “El Mayor” (EM) are 42.7 Km and 62.3 Km from Mexicali, respectively.

The Mexicali Valley (Figure 1) is the third most important agro-industrial region in Mexico. With 180,000 agricultural hectares, it is located in a desert area with an adverse climate, where wheat, corn, cotton, safflower, fodder, and vegetables, are irrigated with water from the Colorado River. Approximately 11,000 farmers harvest three million tons of food a year. Therefore, in recent years, the use of large volumes of pesticides to control pests such as organochlorines, organophosphates, carbamates, pyrethroids, and other inorganic compounds are intrinsically linked to the agro-industrial development of the area [30]. The volunteers for the study are from Ejido Vicente Guerrero (32.3961°, −115.14023°) and Ejido El Mayor (32.12594°, −115.27265°).

### 2.3. Study Groups

The two study groups live under the same environmental conditions in the agricultural Valley of Mexicali in Baja California, Mexico; both groups with different lifestyles and eating habits. The first group was made up of 21 Seventh-day Adventists from Ejido Vicente Guerrero, who practiced a strict LOV diet (rich in fresh vegetables, fruits, eggs, and dairy products), with no tobacco or alcohol consumption, and agriculture as their main economic activity. The second group was made up of 14 Cucapah Indigenous individuals from Ejido El Mayor, whose ancestral custom is a diet low in vegetables, high in fish and shellfish, and eventually, beef and goat meat, cooked with abundant oil, and accompanied mainly by beans, potatoes, and wheat tortillas; their economic activity is focused on agriculture and fishing, and occasionally on crafts and tourism.

### 2.4. Participants Inclusion

Constant communication was established with the community leaders, and through them, the approach to the participants was carried out. Each participant signed an informed consent form and answered a questionnaire to identify their previous history of exposure to contaminants, socioeconomic factors, family inherited conditions, health status, and data such as height, weight, eating habits, alcohol intake, smoking, exercise, and other relevant information. Each participant provided a venous blood sample for L-CBMN assay performed within 24 h after collection.

### 2.5. Selection Criteria

The study groups included participants between 16 and 45 years old with similar lifestyles, diets, and at least one year of residence in the study area. Individuals diagnosed with cancer with or without treatment and those with ongoing or completed medical therapy for at least one month prior to sampling were excluded. Subjects whose samples could not be processed or incomplete information were excluded from the groups [31].

### 2.6. L-CBMN Assay

Cytogenetic stability biomarkers were evaluated based on the cytokinesis-block technique established by Fenech [28]. Briefly, 5 mL of blood was obtained from each participant by venipuncture, which was cultured by duplicate in 15 mL Falcon^®^ tubes (Corning, Glendale, AZ, USA) with 6.3 mL of RPM1-1640 culture medium (Sigma R-8758) supplemented with non-essential amino acids (Sigma M-7145, St. Louis, MO, USA), L-Glutamine (Sigma G-6392, St. Louis, MO, USA), and 0.2 mL of phytohemagglutinin PHA-M (Sigma L-8902, St. Louis, MO, USA). The tubes were incubated at 37 °C for 44 h, stopping proliferation by adding 3µL/mL of cytochalasin-B (Sigma C-6762, St. Louis, MO, USA); after that, the cells were incubated for another 24 h at 37 °C. The lymphocytes were recovered from the culture with a solution of methanol and acetic acid (3:1). After washing, the lymphocytes were stained with eosin (Sigma 318906, St. Louis, MO, USA) and methylene blue (Sigma 03978, St. Louis, MO, USA) and observed under a compound microscope (100×) [32].

### 2.7. Slide Analysis

**Figure 2:**
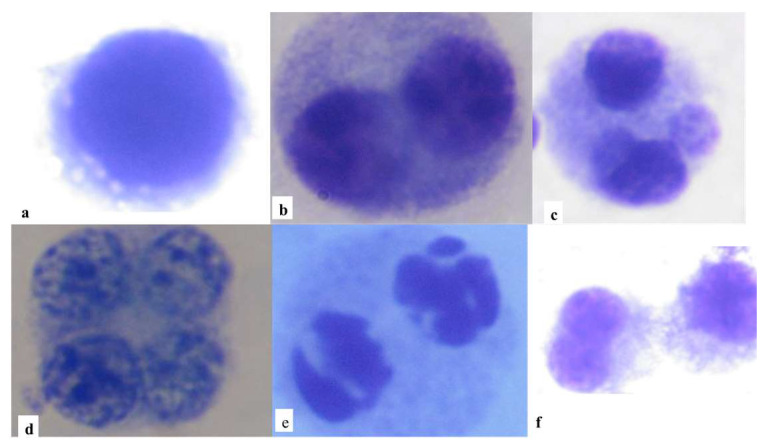
(**a**) Mononucleated, (**b**) binucleated, (**c**) trinucleated, (**d**) tetranucleated, (**e**) apoptotic, and (**f**) necrotic cells stained with eosin and methylene blue. Microphotographs taken with a Carl Zeiss^®^ microscope (Carl Zeiss GmbH, Jena, Germany) with immersion objective (100×).

**Figure 3:**
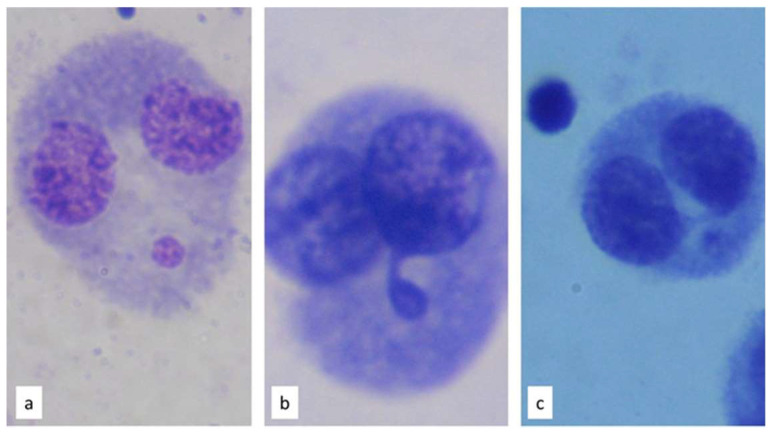
Cells with (**a**) micronuclei (MNs), (**b**) nuclear buds (NBUDs), and (**c**) nucleoplasmic bridges (NPBs) stained with eosin and methylene blue. Microphotographs taken with Carl Zeiss^®^ microscope (Carl Zeiss GmbH, Jena, Germany) with immersion objective (100×).

The cell division index (NDI) was determined for each person based on 500 cells counted. The frequency of necrosis, apoptosis, and mono, bi, tri, and tetranucleated cells were identified as biomarkers of cytotoxicity (Figure 2). To assess genomic instability, 1000 binucleated cells were counted and the frequency of micronuclei (MNs), nuclear buds (NBUDs), and nucleoplasmic bridges (NPBs) was recorded; see Figure 3.

### 2.8. Statistical Analysis

The information was recorded in an Excel© datasheet (Microsoft 365, Version 2307). Data was transformed with square root [31], according to the following equation:

$$X^{\tau }=\sqrt[{2}]{X+\frac{3}{8}}$$

Routine analysis was performed to determine descriptive statistics, the correlation between the six biomarkers using Spearman’s coefficient, the non-parametric statistical analysis of the Mann–Whitney U test, and graphs were generated with GraphPad Prims 9.2©. For statistical analysis, the first group included 14 non-vegetarians from “El Mayor”, and the second group was made up of 19 strictly vegetarian cases from the “Vicente Guerrero” of the original 21 cases. This exclusion was because two of them were pescatarians.

## 3. Results

### 3.1. Socio-Environmental Information

Table 1 collects data on health and socio-environmental variables of the two study communities, including schooling, smoking habits, alcohol consumption, family history of chronic-degenerative diseases, and the frequency consumption of seafood, fruit, and vegetables. The frequency of consumption of these foods, expressed with a Likert scale, suggests a middle- to low-macro and micronutrient uptake in both groups. Full data can be consulted in Supplementary Materials, Table S1. Both groups are overweight according to the average body mass index, which is the highest value of the index for women in both cases of the LOVs and NVs groups. In the LOVs group, more than 70% have secondary education or higher; in contrast, the most frequent educational level was primary and secondary for the NV group (85.6%). A total of 85% of the participants from the LOV group consumed vegetables daily; only 57% of the NV group did. The habitual consumption of fish and shellfish in the NV group was 65%, and 10% of the LOV group declared that they regularly consume fish and seafood. The frequency of history of chronic-degenerative diseases in LOV was 12% and 82% for NV. Finally, 12% of the NV group declared tobacco consumption, 59% consumed alcohol and smoked, and only 29% said no consumption of tobacco or alcohol. In contrast, 100% of the LOV group did not smoke or drink alcohol.

### 3.2. Cytotoxicity and Genotoxicity

As observed in Table 2 and Figure 3, no difference in NDI was found between NV and LOV groups. However, it should be noted that the frequency of apoptosis, necrosis, and all genotoxicity indices (MN, NBUDs, and NPB/1000 cells) in the LOV population was statistically lower compared with the NV group. These differences were detected using the Mann–Whitney U test.

### 3.3. Cytogenotoxic Damage Associated to Substance Abuse

Table 3 shows the average values for cytotoxic and genotoxic biomarkers found in the Cucapah population (NV group). Based on nominal cytogenotoxic biomarkers, tobacco use, but not the combination of tobacco and alcohol, produces the greatest cytotoxic and genotoxic damage compared to those reported without alcohol or tobacco use. It is essential to mention that two-way ANOVA analysis shows statistically significant differences only for apoptosis and NPBs between non-smokers and non-alcohol users and smokers but not alcohol consumers. A similar result was found in apoptosis for tobacco and alcohol and those without alcohol or tobacco use. It is striking that individuals with both habits (smoking and alcohol) present statistically significant differences in apoptosis but not in other cytogenotoxic biomarkers compared to people who do not consume alcohol or tobacco. These results suggest that the consumption of tobacco and alcohol does not represent a significant contribution to the increase in cytogenotoxic biomarkers compared to the non-consumption of these substances in the Cucapah population.

### 3.4. Correlation

**Figure 4:**
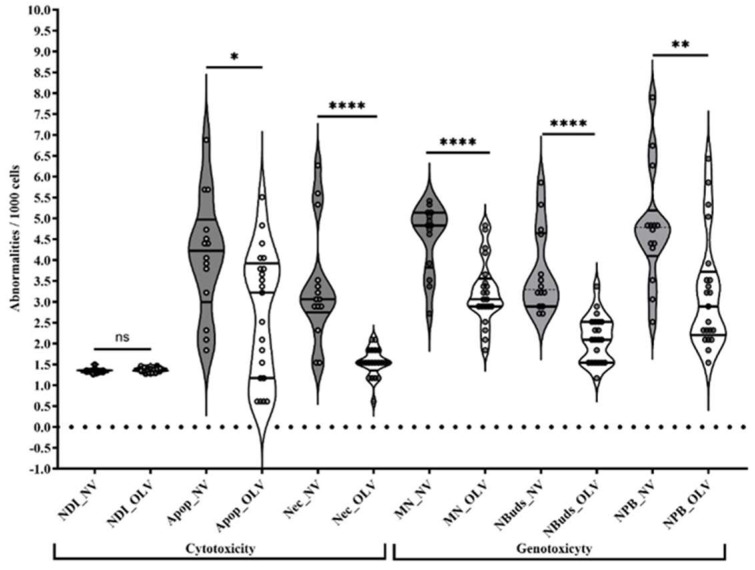
Cytotoxic and genotoxic biomarkers for NV (gray) and LOV (white) groups. NDI—cell proliferation index; Apop—apoptosis; Nec—necrosis, NBUDs—nuclear buds, NPB—nucleoplasmic bridges, MN—micronuclei. Significant differences (* = *p* < 0.05; ** = *p* < 0.01; **** = *p* < 0.0001), ns = non-significative difference using the Mann–Whitney U test.

**Figure 5:**
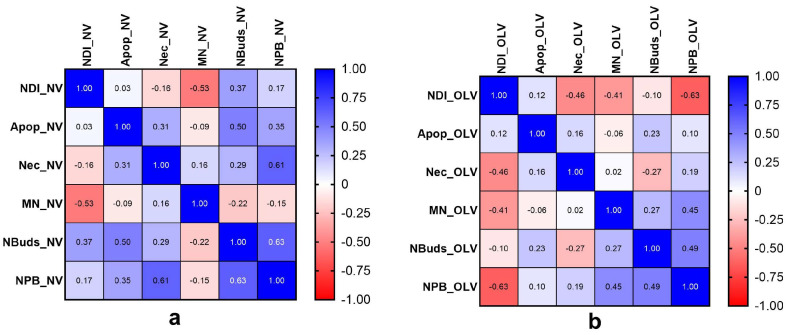
Spearman’s correlation coefficient. (**a**) Correlations of cytotoxic and genotoxic biomarkers of the Cucapah group (NV group) and (**b**) correlations of cytogenotoxic biomarkers for the lacto-ovo vegetarian population (LOV group). Cell division index (NDI), apoptosis (Apop), necrosis (Nec), micronuclei (MN), nuclear buds (NBUDs), and nucleoplasm bridge (NPB). Red indicates negative correlation and blue indicates direct correlation.

Figure 4 shows that NDI is inversely correlated with necrosis and the three genotoxicity biomarkers in the LOV group, being the highest correlation found with MN and NPBs. On the other hand, in the NV group, the highest inverse correlation was between NDI-MN and NDI-necrosis. Therefore, the higher the cell proliferation, the lower the frequency of MN and necrosis in both groups. In addition, a positive association was observed between genotoxic biomarkers MN-NBUDs, MN-NPBs, and NBUDs-NPBs in the LOV group, suggesting a strong correlation between reversible and irreversible genetic damage. Likewise, between NDI-NBUDs, NDI-NPBs, apoptosis-NBUDS, and apoptosis-NPBs in the NV group, the higher the proliferation index and cytotoxic damage, the more significant the number of NBUDs and NPBs in the NV group (Figure 5).

## 4. Discussion

It is essential to maintain health that the genome preserves its integrity and accurately transmits information from progenitor to daughter cells for proper cell function and development. Cells have various complex mechanisms to detect, repair, or eliminate errors to safeguard this integrity. However, it is essential to note that these are not foolproof mechanisms. In addition, continuous exposure to environmental and intracellular genotoxic substances adds risk factors [33]. Environmental and occupational pesticide exposure and other contaminants can cause DNA damage, genomic instability, and oxidative stress [34,35,36]. Furthermore, some reports indicate that several pesticides could be detected in persons, regardless of diet, simply by consuming fruits and vegetables with pesticide residues [37,38,39,40].

Nevertheless, daily habits and diet could contribute to genotoxic damage amelioration, particularly with plant-based diets [17,18,22]. Specifically, the LOV diet excludes red meat, poultry, and fish consumption but includes eggs and dairy products that complete the macro- and micronutrient intake coming from significant amounts of vegetables, fruits, grains, and seeds consumed [41,42]. The LOV diet has been widely studied and documented in the Seventh-day Adventist Church community, identifying a higher life expectancy and lower risk of chronic degenerative diseases than omnivorous populations [12,14,15,43,44,45], despite micronutrient deficiencies, that play critical roles in the antioxidant response of the body [17,23,24,41].

Therefore, the LOV diet could provide significant advantages over environmental or occupational exposure to pesticides that have barely been explored. The objective of this study was to evaluate the frequency of cytotoxicity and genomic instability biomarkers in lymphocytes in two groups of people chronically exposed to pesticides, mainly organochlorines, organophosphates, carbamates, pyrethroids, and other inorganic compounds inherent to the agro-industrial development of the area [30,46]. The first group consisted of Seventh-day Adventists with a strict lacto-ovo vegetarian (LOV) diet, and the second included Cucapah Indigenous people with deficient non-vegetarian (NV) diets.

In this socio-environmental context, the inhabitants of Ejido Vicente Guerrero have higher education than the inhabitants of Ejido El Mayor. This factor could contribute to the observed differences in income (Table 1) and, in turn, in dietary choices [47]. In general terms, it is very precarious for the Cucapah, highlighting that this ethnic group has managed to persist and survive in environmental conditions that are considered adverse [48].

From the biomarkers evaluated (Table 2, Figure 4), it is clear that there is greater cytogenetic health of the practitioners of the LOV diet than the NV group. However, no differences between groups were found in the nuclear division index (NDI), suggesting an acceptable lymphocyte proliferation that is comparable to values reported for healthy individuals and used in this work as Reference Values (Table 2) [28]. At this point it is essential to mention that the cytogenotoxic biomarkers found in LOV practitioners are at least twice those observed in previously reported non-vegetarian individuals without pesticide exposure [32], suggesting damage associated with pesticide exposure without the evidence of cytotoxic damage.

The habits and customs of LOV Adventist practitioners are probably exerting a cytoprotective effect on occupational and environmental exposure to pesticides, as proposed by Fenech and Crott [49], based on the higher intake of folates and flavonoids, identified as potent cytoprotectors against oxidative stress, cytotoxicity, genotoxicity and the formation of MN. This fact could help explain differences in cytogenetic damage observed in the LOV and NV groups studied in this paper. Native NVs, who are most likely deficient in these micronutrients [50], present the highest cytogenotoxic damage agreeing with the evidence that this type of deficiency induces DNA damage, genomic instability, and aneuploidies [49,51,52]. Interestingly, this phenomenon has been observed in clinical and biomonitoring studies in populations regardless of the diet [18,23,25,37].

The literature reports that a combination of tobacco and alcohol consumption represents a higher risk of genotoxic damage and cancer development than individuals who only smoke or consume alcohol [53,54,55]. Interestingly, the cytogenetic damage observed in Cucapah differs from the reported data. In this case, more significant damage was observed in Cucapah individuals who consume tobacco than those who smoke and drink regularly (Table 3). An additional interesting fact is that, except for apoptosis, no statistically significant differences were found in cytogenotoxic parameters between tobacco and alcohol consumers and those who did not consume either (Table 3). The above results could suggest an adaptive response of the body to specific conditions; however, further studies need to be performed to clarify this point.

### Effect of the Exposure to Pesticides on Genotoxicity Biomarkers and the CBMN Cell Death Pathway in the Study Groups

MNs are genotoxicity biomarkers and protagonists of instability and genetic chaos. They contain nuclear-independent DNA caused by anaphase delay and possess unstable membranes prone to rupture without repair. The DNA contained in these structures can condense, replicate, and divide; however, asynchronously to the nuclear genetic material, it can even undergo chromothripsis (massive rearrangement of DNA) and rejoin the nucleus, causing genetic chaos and, with it, cancer [51]. Additionally, if DNA from the micronucleus is released into the cytosol, it triggers the chronic inflammatory response, senescence, and apoptosis [33]. Our results show that the frequency of these structures presents the following pattern: NV > LOV > Reference Value (Table 2). The MN value in the NV group is more than double compared with the LOV group and five times higher than the Reference Values for people without pesticide exposure [32]. Undoubtedly, environmental exposure to pesticides, habits, and customs are determining factors in the formation of MNs, and their biological effects are reflected in the health conditions of each study group.

Under certain conditions, MNs could induce cell death by apoptosis [32], agreeing with the frequency observed in this work, resembling that found for MN with the following pattern: NV > LOV > Reference Value. In the Cucapah (NV group), apoptosis doubled that found in the LOV group and was three times higher than the Reference Values. Similarly, necrosis shows the same pattern as MN and apoptosis. In this case, the frequency of necrosis observed in Cucapah individuals (NV group) is six times higher compared with the LOV group and at least twelve times compared with the Reference Value. It is important to note that NDI values show no differences between NV, LOV, and Reference Values, allowing us to identify greater cytogenetic stability in the LOV group compared with the NV group [23,56].

The observed patterns also identified a genotoxic association with cytotoxic damage, since the greater the cytotoxic damage, the higher the genotoxic biomarkers values, inferred from the association analysis (Figure 3). The biomonitoring carried out in this work reveals that NVs presented more NBUDs than LOV (Figure 2). This biomarker was positively correlated with necrosis and apoptosis in NVs, but that is not observed in the case of the LOV group (Figure 3). The increase in NBUDs in the NV group could be associated with dietary folate deficiencies due to folate deprivation in cultures in human lymphocytes [57,58]. Similarly, the frequency of NPBs was higher in the NV group compared to LOV, with a strong correlation between this biomarker and necrosis, representing a further indication of overall genomic instability for the NV group.

The few studies that report the six biomarkers of genomic instability provided by the CBMN assay limit the comparison of results of the present study. However, some works carried out in Baja California use techniques like micronuclei frequency in buccal mucosa or DNA damage by comet assay in the buccal mucosa. The results of these studies, compiled in Table 4, make it possible to identify an increase in MN increase in a population exposed to pesticides compared to not-exposed individuals; with the exception of the study by Anguiano-Vega et al. [59] that was carried out in Todos Santos analyzing buccal mucosa, where no statistically significant differences were found. The general trend shows a substantial increase in genotoxic damage expressed as higher frequencies of MN, NBUDs, and NPBs in individuals exposed to pesticides compared to non-exposed individuals. These results agree with the trend found in this work where regardless of the diet or daily habits, continuous exposure to pesticides produces more significant cytogenotoxic damage compared to unexposed people.

In summary, people continuously exposed to pesticides but practicing a LOV diet with fresh fruits and vegetables abundance have greater cytogenetic stability and tend to be more protected from environmental and occupational exposure to pesticides than those with an omnivorous diet and low intake of folates from green vegetables [18,25,26,37,49].

## 5. Conclusions

Continuous exposure to pesticides, directly or indirectly, is more likely to develop genotoxic damage that could lead to cancer in exposed than in unexposed people. However, the consumption of a plant-based diet could lessen the observed damage. In this work, the biomonitoring of two populations from the Valley of Mexicali continuously exposed to pesticides shows that regardless of the diet and daily habits, both present cytogenotoxic damage compared to unexposed individuals. Nevertheless, individuals practicing a lacto-ovo vegetarian diet (LOV) exhibit lower damage than those with non-vegetarian habits (NV). Our results suggest that the consumption of green vegetables and fruits improves the intake of folates and antioxidants, which could produce a better antioxidant response that helps reduce genotoxic damage. The LOV diet plays an important role in the cytogenetic stability of persons exposed to pesticides. However, more studies must be performed to obtain conclusive results.

## Figures and Tables

**Table 1:** Socio-environmental variables, diet, and familiar antecedents for lacto-ovo vegetarian (LOV), and non-vegetarian (NV) groups.

Variables	NV n (%)	LOV n (%)
Total	14	21
Men	4 (28.5)	5 (23.8)
Woman	10 (71.5)	16 (76.1)
Average Body Mass Index		
Men	28.0 (28.5)	23.9 (23.8)
Woman	29.9 (71.5)	27.3 (76.1)
Scholar status		
Non	0	3 (14.3)
Elementary	6 (42.8)	2 (9.5)
Middle	6 (42.8)	3 (14.3)
High School	2 (14.3)	10 (47.6)
University	0	2 (9.5)
Average Income *	110	350
No tobacco, no alcohol	5 (29)	15 (71.4)
Tobacco	2 (12)	0
Tobacco and alcohol	10 (59)	0
Diabetics	0	1 (4.7)
Familiar antecedents	14 (100)	12 (57.1)
Fish and seafood intake		
Little or never	5 (35)	19 (90.5)
Regularly/almost daily	9 (65)	2 (9.5)
Fruits and vegetable intake		
Little or never	6 (42.8)	3 (14.3)
Regularly/almost daily	8 (57.1)	18 (85.7)

* Average income expressed in MXN, corresponding to USD 6.47 and USD 20.6 at a currency exchange rate of USD 1 = MXN 16.99 (22 July 2023).

**Table 2:** Cytotoxic and genotoxic biomarkers for LOV and NV groups.

Biomarker			Group	Median	Mean ± s	*p* Value	Significance
Cytotoxicity	NDI		NV	1.4	1.4 ± 0.1	0.1112	NS
			LOV	1.5	1.5 ± 0.1		
			Reference value		1.5 ± 0.003		
	*Cellular dead*	Apoptosis	NV	17.5	18.4 ± 12.2	0.019	*
			LOV	11	9.2 ± 8.62		
			Reference value		5.5 ± 2.1		
		Necrosis	NV	9	13.0 ± 11.2	<0.0001	****
			LOV	2	2.1 ± 1.0		
			Reference value		0		
Genotoxicity	MN		NV	23	20.8 ± 6.8	<0.0001	****
			LOV	9	10.6 ± 5.3		
			Reference value		4.5 ± 0.7		
	NBUDs		NV	10.5	14.3 ± 8.5	<0.0001	****
			LOV	4	4.3 ± 2.4		
			Reference value		7 ± 0		
	NPBs		NV	22.5	24.5 ± 14.9	0.0027	**
			LOV	8	11.8 ± 10.9		
			Reference value		2.5 ± 0.7		

OLV—ovo-lacto-vegetarians (n = 19); NV—non-vegetarians (n = 14); RV—reference value unexposed; NV—[32]; NDI—cell division index (frequency of mono, bi, tri, and tetra-nucleated cells/500 cells); MN—Micronuclei; NBUDs—Nuclear buds; NPB—Nucleoplasmic bridges. Significant differences (* = *p* < 0.05; ** = *p* < 0.01; **** = *p* < 0.0001), using the Mann-Whitney U test.

**Table 3:** Cytotoxic and genotoxic biomarkers values for NV group (Cucapah). Two-way ANOVA ** *p* = 0.015 and *** *p* = 0.0007. The comparison was performed against the No Smoke, No Alcohol group.

Biomarker	No Smoke, No Alcohol (n = 5)	Smoke but Not Alcohol (n = 2)	Smoke and Alcohol (n = 6)
NDI	1.4 ± 0.1	1.4 ± 0.1	1.4 ± 0.1
Apoptosis	15 ± 18	51 ± 44 ***	18 ± 6 **
Necrosis	6.9 ± 8.3	20 ± 3	15 ± 11
MN	21 ± 5	16 ± 13	21 ± 6
NBUDs	12 ± 9	21 ± 18	12 ± 4
NPBs	13 ± 7	41 ± 30 **	23 ± 7

**Table 4:** Evaluation of genotoxic damage in exposed and non-exposed populations to pesticides in different agriculture regions of Baja California, Mexico.

Town	Tissue	Group	n	MN	NBUDs	NPB	Ref
San Quintin	Lymphocytes	NE	15	11	8	4	[59]
		E	25	18	11	8	
		*p*		<0.05	NS	<0.05	
Maneadero	Lymphocytes	NE	22	7.86	6.45	2.80	[60,61]
		E	26	10.15	7.94	2.38	
		*p*		<0.001	0.03	NS	
	Buccal mucosa	NE	73	0.7			[7]
		E	71	4.5			
		*p*		<0.0001	-	-	
Todos Santos	Comet assay (buccal cells)	NE	24	24.51	-	-	[61]
		E	57	46.92	-	-	
		*p*		<0.0001			
	Buccal mucosa	NE	24	58.3			[62]

E—exposed; NE—non-exposed; MN—micronuclei; NBUDs—nuclear buds; NPB—chromatin bridges; *p*—significant differences; NS—not significant difference.

## Data Availability

Not applicable.

## Supplementary Materials

The following supporting information can be downloaded at: https://www.mdpi.com/article/10.3390/toxics11100807/s1, Table S1. Frequency consumption of fruits, vegetables, cereals, dairy, eggs, fish and shellfish, red meat, and chicken expresed with a Likert scale. 5 = very frequently, 4 = frequently, 3 = occasionaly, 2 = rarely, 1 = very rarely, 0 = never.
