# Supplementary material for: Genomic Instability and Cytotoxicity Evaluation of Two Communities Exposed to Pesticides in the Mexicali Valley by the L-CBMN Assay
Source: Toxics. 2023 Sep 25;11(10):807. doi: 10.3390/toxics11100807 (PMC10611012; doi:10.3390/toxics11100807)
Supplement: Supplementary file 1 [file toxics-11-00807-s001.zip › toxics-2557512-supplementary.pdf]

Genomic Instability and Cytotoxicity Evaluation of two communities exposed to pesticides in the Mexicali Valley by the L-CBMN Assay

Balam Ruiz-Ruiz,[1] Olivia Torres-Bugarin,[2*] Erika Zúñiga-Violante,[1] Francisco Casillas-Figueroa,[1] Roberto Luna-Vázquez-Gómez,[1] Verónica Campos Gallegos,[1] Ana Erika Ruiz-Arellano,[3] María Evarista Arellano-García[1*]

[1] Laboratorio de Genotoxicología Ambiental, Facultad de Ciencias, Universidad Autónoma de Baja California, Ensenada 22860, Baja California, Mexico; bruiz@uabc.edu.mx, casillas.francisco@uabc.edu.mx, rluna@uabc.edu.mx, evarista.arellano@uabc.edu.mx
[2] Laboratorio de Evaluación de Genotóxicos. Medicina Interna II. Facultad de Medicina. Decanato de Ciencias de la Salud. Universidad Autónoma de Guadalajara, Zapopan 45129, Jalisco, Mexico; oliviatorres@hotmail.com
[3] Facultad de Ingeniería, Arquitectura y Diseño, Universidad Autónoma de Baja California, Ensenada 22860, Baja California, Mexico; e-mail@e-mail.com

* Correspondence: O.T.-B. oliviatorres@hotmail.com; Tel.: (optional; include country code; if there are multiple corresponding authors, add author initials), M.E.A.-G. evarista.arellano@uabc.edu.mx

Supplementary Material

Table S1. Frequency consumption of fruits, vegetables, cereals, dairy, eggs, fish and shellfish, red meat, and chicken expresed with a Likert scale. 5=very frequently, 4= frequently, 3=occasionaly, 2=rarely, 1=very rarely, 0= never.

Location	Sex	Fruits	Vegetables	cereals	dairy	egg	fish and shellfish	red meat	chicken
EM	F	3	2	4	4	4	2	2	4
EM	F	2	4	4	4	4	3	4	4
EM	F	3	3	4	4	4	3	3	4
EM	M	3	2	4	4	2	1	3	3
EM	F	4	2	4	2	4	4	4	4
EM	F	2	3	4	3	4	2	3	3
EM	F	4	4	4	4	1	1	4	4
EM	M	2	2	4	1	4	2	2	3
EM	F	3	3	4	3	3	3	3	4
EM	M	1	3	4	2	1	3	2	3
EM	F	4	3	3	2	4	4	4	4
EM	F	3	3	4	3	4	2	3	3
EM	F	3	4	4	2	4	3	3	3
EM	M	3	3	4	3	3	3	3	3
EVG	F	3	2	4	3	4	1	1	1
EVG	F	2	2	3	4	3	2	0	3
EVG	F	3	4	4	1	2	2	1	2

EVG	F	4	4	3	2	1	1	1	1
EVG	M	3	3	4	3	3	2	1	3
EVG	M	2	4	4	3	4	1	1	3
EVG	F	2	4	4	3	4	1	1	3
EVG	M	2	3	4	4	2	1	1	3
EVG	F	2	2	2	1	3	1	1	3
EVG	F	3	3	3	3	3	2	0	3
EVG	F	2	3	4	3	1	3	0	3
EVG	F	4	4	4	4	2	1	1	1
EVG	F	2	4	2	2	1	1	1	1
EVG	F	3	3	4	3	3	2	0	2
EVG	F	3	2	4	4	2	3	0	3
EVG	F	4	3	2	3	2	2	0	3
EVG	F	1	3	3	1	4	1	1	3
EVG	M	3	3	3	3	4	2	0	2
EVG	F	4	4	4	3	3	2	1	2
EVG	F	2	3	4	2	3	1	0	1
EVG	M	3	3	4	1	4	1	1	1
